# Visualized simulative surgery in preoperative planning for proximal femoral varus osteotomy of DDH

**DOI:** 10.1186/s12891-022-05219-7

**Published:** 2022-03-28

**Authors:** Wen Zhou, Haoyu Guo, Rongjie Duan, Qiang Shi

**Affiliations:** 1 Clinical Nursing Teaching and Research Section, the Second Xiangya Hospital, Central South University, Changsha, 410011 China; 2 Department of Infectious Disease, the Affiliated Changsha Central Hospital, Hengyang Medical School, University of South China, 161 Southern Shaoshan Road, Changsha, 410004 China; 3 Department of Sports Medicine, Xiangya Hospital, Central South University, Changsha, 410008 China; 4 Department of Spine Surgery, the Affiliated Changsha Central Hospital, Hengyang Medical School, University of South China, 161 Southern Shaoshan Road, Changsha, 410004 China

**Keywords:** Developmental dysplasia of the hip, Visualized simulative surgery, Osteotomy, Computer-aided design, Three-dimensional reconstruction

## Abstract

**Purpose:**

To assess the preoperative planning of visualized simulative surgery (VSS) and clinical outcomes based on computer-aided design (CAD) and 3D reconstruction for proximal femoral varus osteotomy of DDH.

**Methods:**

A total of 31 consecutive patients (23 females and 8 males) with DDH who underwent proximal femoral varus osteotomy were retrospectively reviewed between June 2014 and July 2018. Patients were divided into conventional group (*n* = 15) and VSS group (*n* = 16) according to different surgical methods. In VSS group, 16 consecutive patients who underwent proximal femoral varus osteotomy were evaluated preoperatively with the aid of VSS. The VSS steps included morphological evaluation of DDH, simulated reconstruction of proximal femoral varus osteotomy, and the implantation of locking compression pediatric hip plate (LCP-PHP). Meanwhile, the osteotomy degrees, surgery time, and radiation exposure were compared between the two groups.

**Results:**

The average follow-up time was 33.5 months (range, 24 to 46 months). The varus angle for proximal femoral varus osteotomy was 24.2 ± 1.1° in VSS group and 25.1 ± 1.0° in conventional group (*P* = 0.4974). The surgery time was 31.0 ± 4.5 mins in VSS group and 48.2 ± 7.3 mins in conventional group, while radiography was 5.0 ± 1.5 times in VSS group and 8.3 ± 2.4 times in conventional group. There was a statistical significance in surgery time and radiography (*P* <  0.0001) when compared with the conventional group.

**Conclusion:**

The VSS can greatly decrease surgery time and radiation exposure for proximal femoral varus osteotomy, which could also be a tool to train young doctors to improve surgical skills and academic communication.

**Supplementary Information:**

The online version contains supplementary material available at 10.1186/s12891-022-05219-7.

## Background

Proximal femoral varus osteotomy with locking compression pediatric hip plate (LCP-PHP; Synthes, Switzerland) is an effective surgical procedure for DDH, which could provide a stable and concentrical hip joint in children via decreasing excessive neck-shaft angle [1–3]. Nevertheless, an ideal surgery is still a technical challenge for most orthopaedic surgeons because inappropriate surgical management may result in severe complications such as re-dislocation or avascular necrosis (AVN) [4, 5]. The success of the surgery mainly depends on the surgeon’s experience, which includes both excellent preoperative planning and precise intra-operative design. Meanwhile, radiography and CT images (including 2-D and 3-D imaging) were not sufficient to provide accurate information about complex deformities of the proximal femur in patients with DDH [6]. Moreover, individual differences in the deformed femoral neck could also affect the accuracy of clinical outcomes [7]. Therefore, a more precise, simple, and effective method is needed for proximal femoral varus osteotomy of DDH.

Precise preoperative planning play a vital role in the field of orthopaedics, which can provide radiological evaluation and detailed information before surgery [8]. With the advances in computer-assisted technology, visualized simulative surgery (VSS) has been demonstrated as a valuable tool in total hip arthroplasty [9], which also gradually becomes popular for detailed three-dimensional preoperative planning in trauma surgery [10–13]. Zheng et al. designed a 3D-printed navigation template for the proximal femoral varus rotation and shortening osteotomy and achieved good results [6]. However, no preoperative planning with the aid of VSS for proximal femoral varus osteotomy has been reported.

Based on computer-aided design (CAD) and 3D reconstruction, the present study was aim to assess the clinical outcomes of VSS for preoperative planning for proximal femoral varus osteotomy of DDH.

## Methods

### Patients’ information

A total of 31 consecutive patients (23 females and 8 males) with DDH who underwent proximal femoral varus osteotomy were retrospectively reviewed between June 2014 and July 2018. According to different surgical methods, 15 patients treated by proximal femoral varus osteotomy were divided into the conventional group in the initial period, then 16 patients treated by proximal femoral varus osteotomy with the aid of VSS technology were divided into VSS group. Meanwhile, hip subluxation or classified as Tönnis grade I was excluded in this study. We collected the CT and clinical data from medical image database in our hospital. Power calculation was used in all cases of two groups. The study protocol was approved by the Institutional Review Board of the authors’ institution. Informed consent was obtained from all their parent before carrying out any research work. All methods were carried out in accordance with relevant guidelines and regulations for human.

### Visualized simulative surgery

**Fig. 1:**
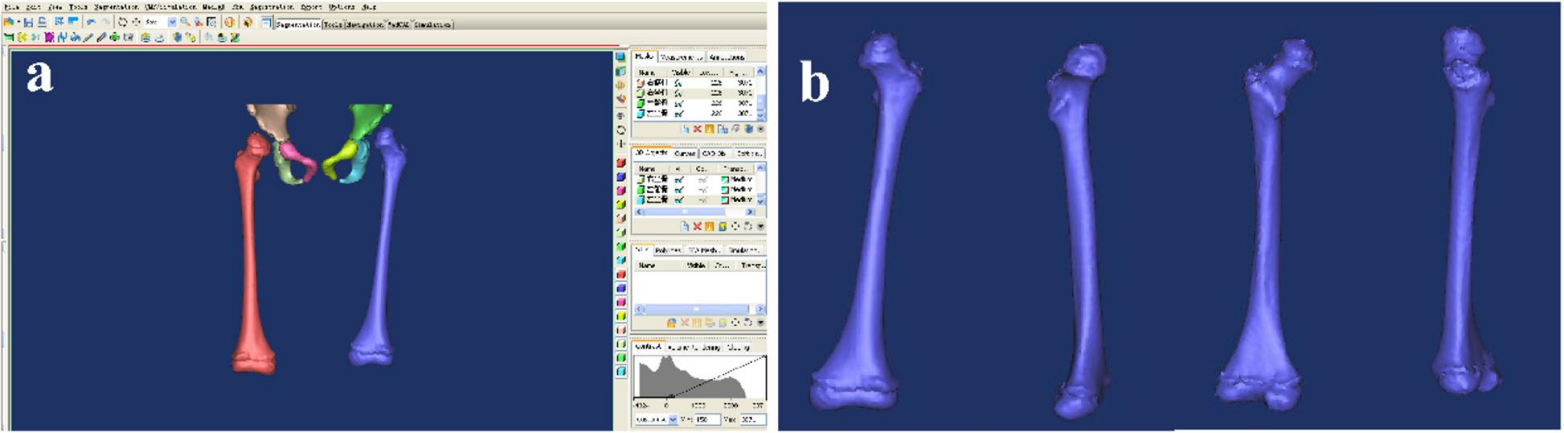
The preoperative 3-D reconstruction for DDH. **a** The 3-D image of DDH was reconstructed via Mimics. **b** Observation and analysis of the abnormal femur in all directions

**Fig. 2:**
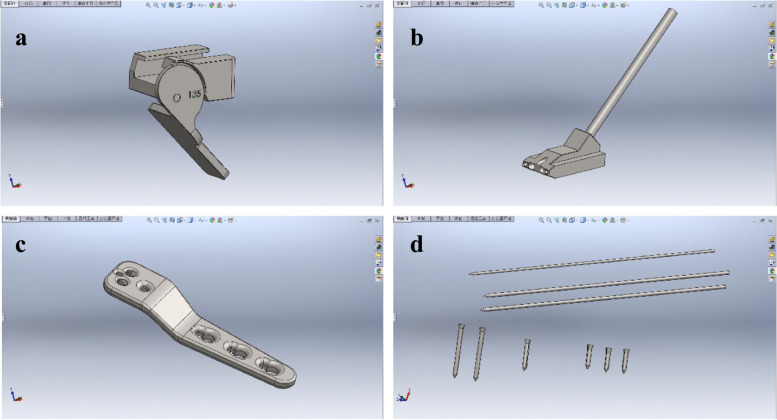
Construct virtual surgical instrument bank. **a** Positioner for aiming device. **b** Aiming block. c Varus plate 3.5 mm 110°. d Kirschner wires and screws

**Fig. 3:**
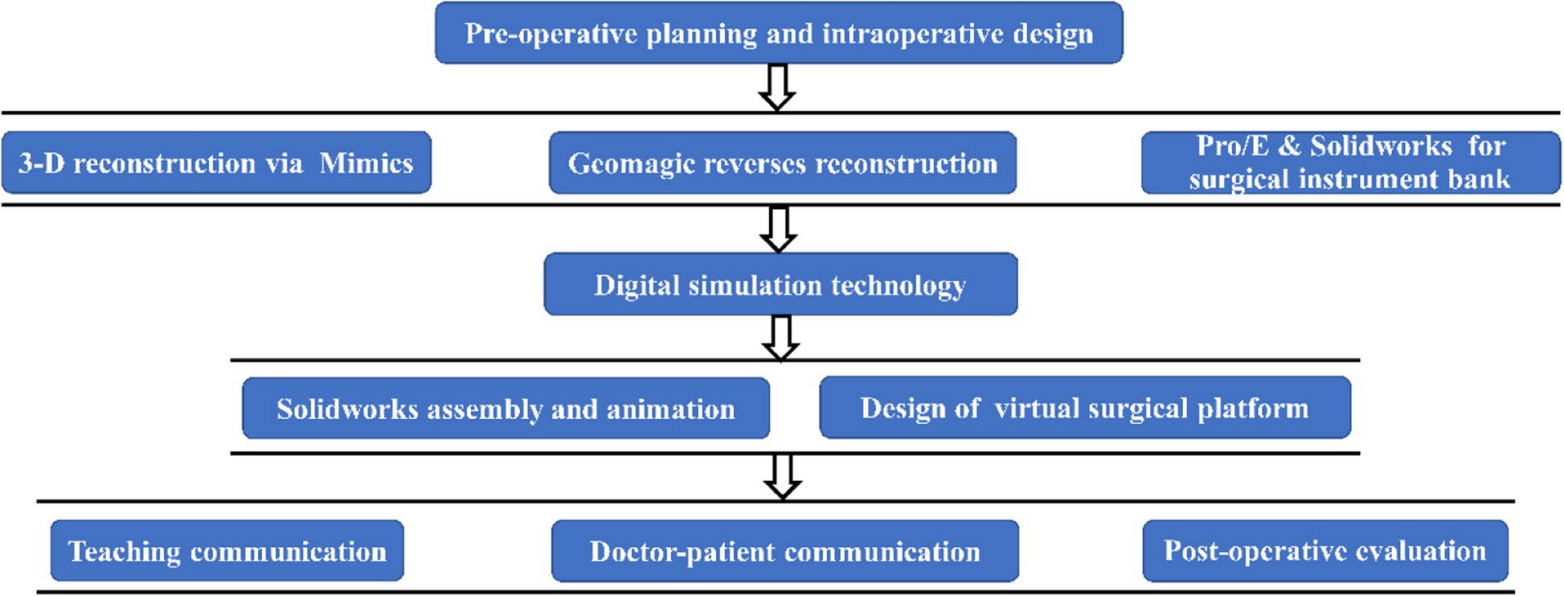
Flow diagram of visualized simulative surgery for proximal femoral varus osteotomy

**Fig. 4:**
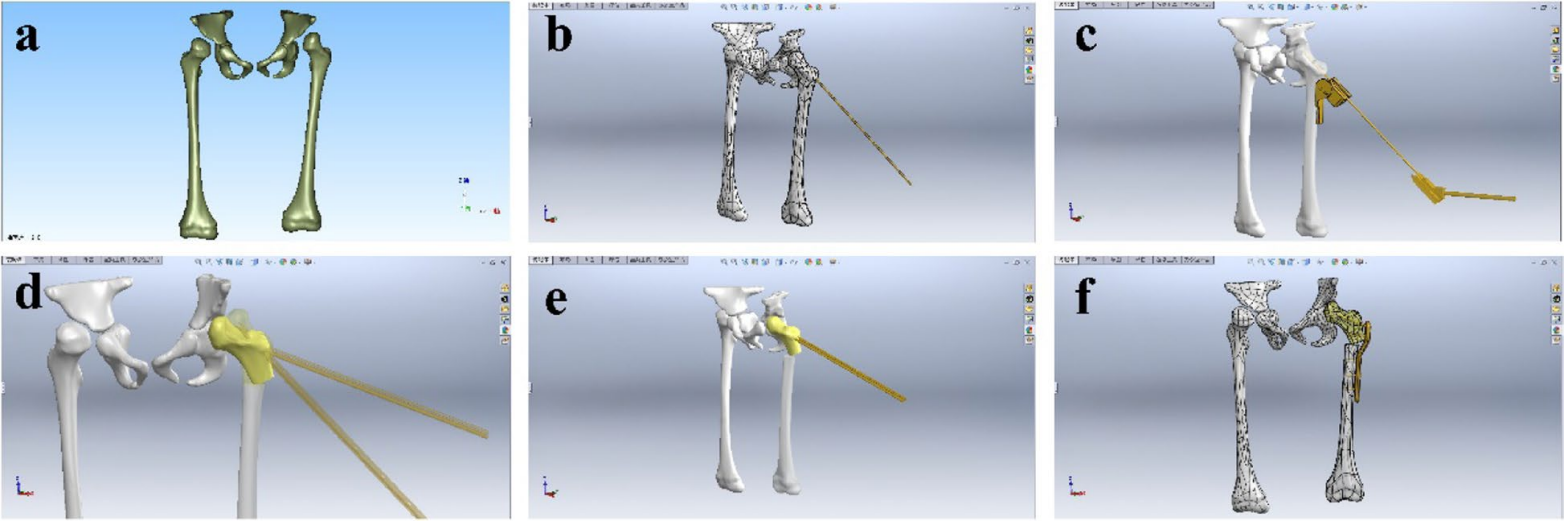
The VSS for preoperative planning of proximal femoral varus osteotomy. **a** Geomagic reverses reconstruction of DDH. **b** Place a 2.0 K-wire above the ventral surface of the femoral neck, which marks the anteversion of the femoral neck. **c** Apply the aiming block for 3.5 mm screws with help of the positioner for aiming device, in order to place the central guide wire approximately 10 mm distal to the physis of the greater trochanter. **d** Calculate the varus angle via Solidworks design software. **e** Simulate proximal femoral varus osteotomy. **f** The osteotomy was performed with LCP-PHP as preoperative planning

The CT scanning data were input into Mimics 20.0 software (Materialise, Leuven, Belgium) for 3-D reconstruction, which could be useful to observe and analyze deformity of the proximal femur in all directions (Fig. 1). Next virtual surgical instrument bank was constructed using SolidWorks 2015 software (SolidWorks Corp) (Fig. 2). Using the VSS, proximal femoral varus osteotomy was performed for preoperative planning as follows (Fig. 3). The correction angles for varus and rotation were determined in the preoperative image evaluation according to the contralateral parameters (unilateral DDH) or normal parameters (bilateral DDH) in both the VSS and the conventional groups. After the digital model of the proximal femur was input onto a reverse engineering software Geomagic Studio by the format of STL, the desired neck-shaft angle was determined and varus rotation was designed via preoperative virtual surgical protocol formulation. Place one K-wire above the ventral surface of the femoral neck on the computer, which marks the anteversion of the femoral neck. Then apply the aiming block for screws with the help of the positioner for the aiming device. After that, the proximal femoral varus osteotomy with LCP-PHP was simulated as preoperative planning (Fig. 4). The whole procedure using the VSS was presented as animation (Video).

### Surgical procedures and post-operative management

**Fig. 5:**
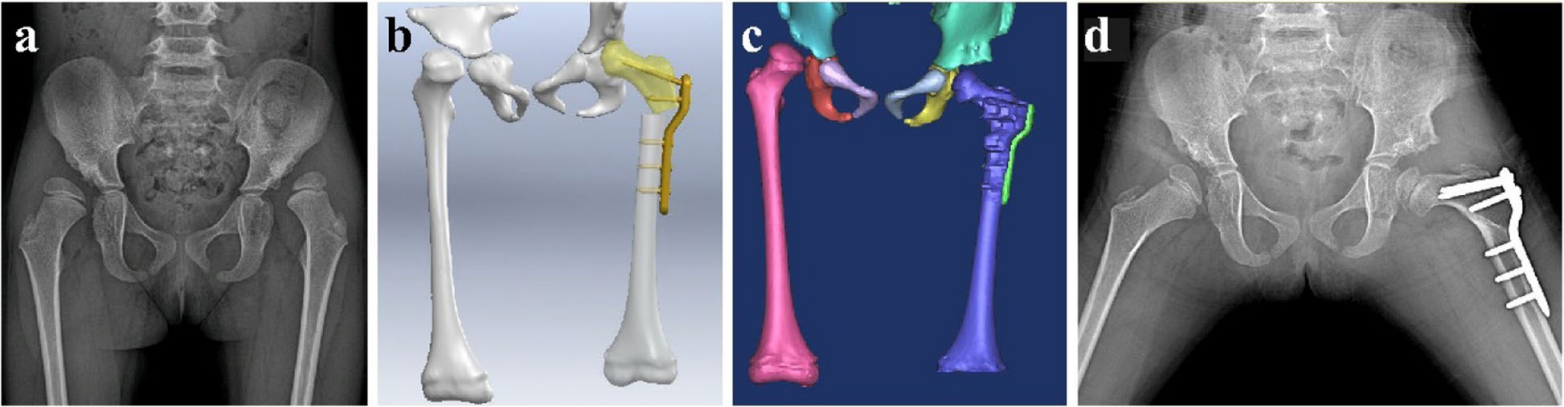
Radiographs for proximal femoral varus osteotomy via VSS. **a** The preoperative anteroposterior radiograph indicated that bilateral DDH occurred in a 4-year-old girl. **b** The proximal femoral varus osteotomy with LCP-PHP was simulated via VSS preoperatively. **c** The 3-D image of pelvis and femur were reconstructed via Mimics on the first postoperative day. **d** The post-operative radiograph showed anatomical correction and good appearance at 13 months of follow-up

The proximal femoral varus osteotomy with LCP-PHP was performed by one senior orthopaedic surgeon in our department (Fig. 5). In VSS group, an experienced orthopaedic doctor performed simulated surgery on the computer preoperatively according to the DDH patients’ condition. With the aid of VSS, the surgeon can calculate the varus angle and perform proximal femoral varus osteotomy precisely. For example, the desired rotation angle was 15° in femoral varus for concentric reduction between femoral head and acetabulum, which was designed by an experienced orthopaedic doctor via VSS. Meanwhile, the control group performed the proximal femoral varus osteotomy with freehand manner. The post-operative neck-shaft angle, surgery time, and radiation exposure were recorded. No significant difference in post-operative management procedures between the two groups. A hip spica cast was used for 8 weeks and a double lower limb brace with hip abduction for another 8 weeks. Moreover, radiographs were taken at 2, 4 and 6 months and then every year until skeletal maturity.

### Statistical analysis

The data in this study were statistically analyzed via SPSS 25.0 software (SPSS, Inc., Chicago, USA) and manifested as count (percentage) or mean ± standard deviation (SD). Student’s t-test, chi-squared test, and Fisher’s exact test were also used. Different parameters measured between two groups were assessed with an independent t-test for continuous variables, and a chi-square test or Fisher’s exact test for the categorical variables. *P* <  0.05 was considered to be statistically significant.

## Results

The average follow-up time was 33.5 months (range, 24 to 46 months). In VSS group, post-operative radiographs showed excellent results at the final follow-up. On the first postoperative day, the varus angle for proximal femoral varus osteotomy was 24.2 ± 1.1° in VSS group and 25.1 ± 1.0° in conventional group (*P* = 0.4974). The surgery time was 31.0 ± 4.5 mins in VSS group and 48.2 ± 7.3 mins in conventional group, while radiography was 5.0 ± 1.5 times in VSS group and 8.3 ± 2.4 times in conventional group. One case suffered with epiphyseal growth plate violation by the implant in conventional group. There was a statistical significance in surgery time and radiography (*P* < 0.0001) when compared with the conventional group (Table 1). It is proved that the sample size of the two groups was sufficient when the Power calculation greater than 0.9.

**Table 1:** Comparison of operation data and functional outcomes

	Conventional group (*n* = 15)	VSS group (*n* = 16)	*P* value	Power calculation
Osteotomy degrees, °	25.1 ± 1.0°	24.2 ± 1.1°	0.4974	0.63740
Surgery time, mins	48.2 ± 7.3	31.0 ± 4.5	< 0.0001	1.00000
Radiography, times	8.3 ± 2.4	5.0 ± 1.5	< 0.0001	0.99205
McKay standard, n (%)			0.1441	
Excellent	8	14		
Good	4	1		
Fair	1	1		
Poor	2	0		

It is proved that the sample size of the two groups was sufficient when the Power calculation greater than 0.9

Based on the McKay criteria [14], 14 patients in VSS group obtained excellent correction, 1 patient obtained good prognosis, and 1 patient obtained a fair prognosis, while 8 patients were scored as excellent, 4 as good, 1 as fair, and 2 as poor in conventional group. In a word, the results between the two groups were no significant difference (*P* = 0.1441).

## Discussions

The goal of ideal corrective surgery for DDH in children is to achieve concentric reduction of the hip and avoid avascular necrosis (AVN), which is a technical challenge for orthopaedic surgeons [15–18]. It is now well accepted that one of the most effective strategies for the management of DDH is proximal femoral varus osteotomy with LCP-PHP [19]. However, it is more challenging to perform the surgery into the narrow proximal femur for DDH patients. Therefore, preoperative planning for proximal femoral varus osteotomy is very crucial. The main finding of this study was that the VSS can greatly decrease surgery time and radiation exposure for proximal femoral varus osteotomy. In a word, this is the first study to assess the preoperative planning of VSS and clinical outcomes for proximal femoral varus osteotomy of DDH.

With the development of orthopaedic instruments, LCP-PHP has become widely used to reach a more precise angular correction with locking screws [20]. However, implantation of LCP-PHP requires a long learning curve. Firstly, individual differences in proximal femoral size, the neck-shaft angle, and the femoral anteversion add to the difficulty of the implantation of LCP-PHP, and thus preoperative calculation and measurement of these angles for individualized surgical design are essential. In the conventional group, the neck-shaft angle was assessed on pelvic X-ray while femoral anteversion was measured via spiral CT scan and three-dimensional reconstruction of the femur using specific scan parameters (120 kV; 120 mAs; 1-mm-thick slices, 0.5 mm interlamellar spacing). Besides, the pin angle and depth of LCP-PHP intra-operatively need to be adjusted several times to achieve ideal position, which may prolong the operative time and increase the risk of iatrogenic vascular and nerve injury. Finally, X-ray or CT was not sufficient to provide accurate information for preoperative simulation or intra-operative design of DDH. Therefore, new techniques for implanting LCP-PHP are urgently needed, which must be practical, precise, and easy to promote.

Various navigation templates using 3D printing technology have been applied for the treatment of DDH in children, however, more bone markers are needed to increase the degree of fitting and the stability of the plate [3, 6, 7, 21]. Currently, the virtual surgical protocol formulation for preoperative planning of DDH was few reported [8]. Therefore, in the present study, we used VSS to improve the efficacy and precision of proximal femoral varus osteotomy with LCP-PHP in DDH. Based on CAD and 3D reconstruction, the advantages of VSS for proximal femoral varus osteotomy with LCP-PHP are as follows: (1) The orthopaedics surgeon can observe the pathological conditions of the proximal femur clearly in every direction and measure the excessive neck-shaft angle and femoral anteversion accurately before surgery. Meanwhile, the preoperative surgical plans and proximal femoral varus osteotomy with LCP-PHP can be simulated and modified. (2) The virtual surgical protocol formulation can help the orthopaedics surgeon perform the surgical operation process proficiently and make the intra-operative design appropriately, which can not only reduce the radiation exposure and operation time, but also improve the accuracy and safety of the surgery. (3) Fully understanding proximal femoral varus osteotomy with LCP-PHP via VSS can help parents understand their children’s medical condition while promoting doctor-patient communication. Moreover, it could also be a tool to train young doctors to improve surgical skills and academic communication for proximal femoral varus osteotomy.

Nevertheless, there were some limitations in the present study. First, more cases are required to evaluate the accuracy and efficacy of VSS for DDH because no significant difference was demonstrated for clinical outcomes or correction angle between the VSS and conventional groups. Besides, simulated operation on the 3D printing model or patient-specific instrument should be performed in future studies, which could increase its clinical value in orthopaedics surgery. Second, discrepancy in the collection angles between the preoperative planning and postoperative CT measurement and comparison of the epiphyseal growth plate violation rate between the groups should be included in the comparative analysis. Third, the femoral anteversion angle was also crucial for proximal femoral varus osteotomy and it would be better to present the data of the femoral anteversion angle before and after surgery during the VSS surgery. Meanwhile, the VSS could not be only used for proximal femoral varus osteotomy, the pelvic osteotomy or other pediatric hip diseases will also be taken into account to assess the VSS’s effect in our following study.

## Conclusion

The VSS based on CAD and 3D reconstruction can greatly decrease surgery time and radiation exposure for proximal femoral varus osteotomy, which could also be a tool to train young doctors to improve surgical skills and academic communication.

## Supplementary Information

**Additional file 1.**

## Data Availability

The datasets used and analyzed during the current study are available from the corresponding author on reasonable request.

## Abbreviations

VSS: Visualized simulative surgery
CAD: Computer-aided design
AVN: Avascular necrosis
LCP-PHP: Locking compression pediatric hip plate
DDH: Developmental dysplasia of the hip
3D: Three-dimensional
